# Men’s experience of perpetrating intimate partner violence following disclosure of HIV status by their seropositive female intimate partners: a qualitative study

**DOI:** 10.1080/07853890.2022.2062444

**Published:** 2022-05-05

**Authors:** Felix Apiribu, Sinegugu Evidence Duma, Busisiwe Purity Ncama

**Affiliations:** a School of Nursing and Public Health, College of Health Sciences, University of KwaZulu-Natal, Durban, South Africa; b Department of Nursing, Faculty of Allied Health Sciences, College of Health Sciences, Kwame Nkrumah University of Science and Technology, Kumasi, Ghana

**Keywords:** Heterosexual, HIV seropositive, intimate partner, perpetrator, views

## Abstract

**Background:**

Gender-Based (GB) intimate partner violence is a social and public health issue globally. Several risks of violence related to male sexual partners’ perpetration of intimate partner violence (IPV) following the disclosure of their female intimate partners’ HIV + status have been reported. No research has been conducted on male sexual partner’s perspectives of perpetrating IPV following their female intimate partners’ disclosure of human immunodeficiency virus (HIV) seropositive status as a risk factor for the perpetration of IPV in Ghana.

**Objective:**

The objective of this study is to explore and describe male sexual partners’ views or perspectives of perpetrating IPV following their female intimate partners’ disclosure of being HIV positive in Ghana.

**Methods:**

Interpretive phenomenological approach was used to collect and analyse data from a purposive sample of 18 Male participants whose female intimate relations informed them of being HIV + in Ghana. The sample population was taken from Ghana because such research has been reported elsewhere but none has been done in Ghana. A semi-structured interview guide was used to collect the data. The interview guide covered topics such as background information, participants’ reaction to HIV positive disclosure, lived experiences of participants, and Participants’ understanding of different forms of IPV.

**Results:**

The findings of this study reveal five main themes that emerged from the interviews which include views on the perpetration of emotional, psychological, and verbal abuse; views on the perpetration of sexual deprivation; views on the perpetration of social isolation; views on the perpetration of financial abuse and views on escalated perpetration of physical abuse.

**Conclusion:**

From the data, HIV positive status disclosure served as a risk factor for different forms of GB IPV against HIV positive women in Ghana, thus making this group more vulnerable and exposed to more GB IPV. Strategies to prevent the perpetration of IPV against women newly diagnosed as HIV positive are needed. We recommend screening all newly diagnosed HIV-positive women for abuse as an additional prevention strategy for IPV associated with disclosure of positive HIV status.

**KEY MESSAGES**
- HIV positive status disclosure serves as a risk for the perpetration of IPV.
- Men are predisposed to violence upon hearing that their female heterosexual intimate partners are HIV positive.
- HIV infection information is distressful to receive from an intimate partner.

## Background

Intimate partner violence (IPV) is a major public health problem for victims and society as a whole. The perpetrators of IPV are mostly men and the victims or survivors are mostly women in Ghana [1,2]. The perpetration of IPV by men is responsible for one-third of murders of women, globally [1,3,4]. Risky behaviours like drug use and male dominance are related to persistent domestic violence (DB) perpetrated by men [5]. It has been reported that there is a positive association between IPV and HIV risk behaviours and HIV infection globally [6]. This means that IPV can lead to increased risky behaviours like lack of use of condoms and forced sexual encounters which will subsequently lead to increased HIV infections. This highlights the need for public health preventive measures to be taken [7]. These measures include education on the use of condoms, behaviour change strategies, and the early identification of IPV.

Research conducted in the United States estimates that nearly one in five men in that country are reported to have perpetrated IPV towards an intimate partner [6]. Similar national estimates have been reported in African countries, including Liberia, Sierra Leone, Burkina Faso, Ghana [8,9]. Evidence generally shows a high (61%) prevalence rate of physical IPV in the global population [10] with more than 50% of this occurring in Sub-Saharan Africa (SSA) [10].

Additionally, several risks factors related to male perpetration of violence to their spouses have been reported, but limited research has been conducted on male sexual partner’s perspectives of perpetrating IPV following their female intimate partners’ disclosure of being HIV seropositive. There is limited empirical data regarding the reasons or motives for the male sexual partner’s perpetration of intimate partner violence against their female intimate partners when they disclose their HIV seropositive status [2,11].

Women infected with HIV encounter several risks factors and susceptibilities, and violence is among these risk factors when one is infected with the disease. For instance, the power struggle in intimate relationships is positively correlated to the failure to negotiate safe sex and/or use condoms as protection [12,13]. Gender roles and inequity are pervasive given the perceptions of the traditional male-dominant role and behaviours that lead to violence in their current or previous relationships in Sub-Saharan Africa (SSA) [14]. Several efforts have been put in place to minimise stigma and discrimination against people living with HIV (PLWHIV) globally. However, evidence suggests that HIV-positive women are at an increased risk of going through IPV perpetration resulting from the information of their HIV + status to their male relations [15]. If not addressed, this risk factor for Gender-Based IPV following disclosure of seropositive HIV status may reverse all the gains that have been made in minimising the stigma and discrimination against PLWHIV.

A study conducted in Tanzania found that women infected with HIV were significantly more likely to have had a physically violent male partner at some time and to have experienced physical and/or sexual violence with a current or previous partner [16]. Previous studies have indicated that pieces of evidence of an increased HIV + population of women in some African countries might have had an increased risk of IPV as a result of their HIV + Statuses when they informed their male intimate relations [17,18]. Some issues in relation to IPV will likely be aggravated in some settings or countries with deprived resources, including Ghana, where there is increased discrimination and stigmatisation. HIV-positive women in such settings risk being disliked, deprived of some vital services, censured, sacked from their homes, or even divorced. They may even be accused of causing the disease in the family or even in the communities [17]. Shamu et al. [17] identified about forty percent of HIV + women who were pregnant in Zimbabwe were abused when they disclosed their HIV + statuses to their intimate male relation.

In Ghana and other African countries, HIV-positive status is usually diagnosed at the ante-natal clinics (ANC) because it is mandatory to test pregnant women for HIV [19,20]. Women’s fear of their partners’ negative responses and perpetration of IPV following disclosure of seropositive status may result in non-disclosure of a seropositive HIV status to their intimate partners. This may then decrease HIV prevention behaviours such as the use of condoms. Non-disclosure of seropositive HIV status increases the risk of infecting one’s partner with HIV [17,20]. Ezebuka et al. [21] confirm that the disclosure of positive HIV status increases the risk of IPV against women living with HIV (WLWHIV) by their male partners. In their findings, the post-disclosure IPV rate was significantly higher than for pre-disclosure and was greater than the national domestic violence rate in Nigeria. The post-disclosure IPV rate was strongly correlated with the HIV-positive status of the male partner and the multiplicity of sexual partners.

Exploring male sexual partners' perspectives of their own perpetration of violence when their female HIV + partners informed them about their infection statuses is very important due to the serious implications this can have on women's wellbeing. The objective of this paper is, therefore, to explore and describe the experiences of men who perpetrated violence when their female partners had informed them of being infected with the virus.

## Methods and materials

### Research design

The researchers employed an interpretive phenomenology to explore and describe male sexual partners’ perspectives of perpetrating IPV after their partners had been informed of being HIV seropositive in Ghana. This was the most suitable approach for the researchers to gain an in-depth understanding of the lived experiences of the perpetrators on visiting violence on their intimate relations and their partners informing them of their infection status [22]. The research design used in this study was an exploratory qualitative one. An interpretive phenomenology was used in the collection of the data. The qualitative research design was chosen because of the sensitive nature of this study and this method has been identified as the most suitable method for such studies [23,24]. Interpretive phenomenology was also used because it was concerned with the interpretation of the structures of experience and how things are understood by people who live through these experiences and by those who study them [25–28]. Heidegger introduced the concept of dasein (the human way of being in the world) to emphasise that individuals cannot detach themselves from various contexts that influence their choices and give meaning to lived experiences. Dasein, Heidegger says, is the entity “we ourselves are”, the entity usually labelled the “subject” or the “human being.” In other words, Dasein is an *ontic* notion, it does not denote being or a mode of being, but a particular entity. It does so, however, in a formally indicative way [29–31]. Therefore, Heidegger’s phenomenology attempts to address the situatedness of an individual’s human way of being in the world (dasein) about the broader social, political, and cultural contexts [10]. Therefore, when we consider what is it like to experience caring, healing, and wholeness we cannot ignore the lives people live outside of being ill or well. This study, therefore, made use of the lived experiences of the participants in the context in which they find themselves using explorative qualitative research design.

### Study setting

The setting where this study was done was in two HIV clinics, one of which is a tertiary health institution and a district hospital in Ghana respectively.

### Target population and sampling

To participate in the research, participants were enlisted as part of the study if they were older than 18** **years. Men who admitted to having been abused physically or intellectually, emotionally were recruited. They were recruited if they were deprived of sex or financially following their intimate partner’s disclosure of being HIV positive. Also, where women reported an escalation of abuse since disclosure of seropositive HIV status^1^ by their male partners were also included. The study excluded men who abuse their female partners but indicated that they had access to weapons. Men who were abusive, but were seriously ill and could not speak for themselves were excluded.

The sample of eighteen men was reached through data saturation. Data saturation is when no new information seems to emerge during coding, that is, when no new properties, dimensions, conditions, actions/interactions, or consequences are seen in the data [32] In line with this, there was no new information, no new properties, dimensions, actions/interactions, or consequences after the 18th participant.

The target population was men who perpetrated violence against their female intimate relations who currently disclosed their HIV + statuses. Purposive sampling technique was used to recruit a sample of eighteen participants for the study. The researchers visited the two settings (recruitment link)^i^ where HIV + men and women receive routine chronic HIV health care management. Women who were approached and asked for their permission to assess them for abuse were women who were assessed for possible abuse by their male intimate partners. Those who were found to be within the criteria for inclusion were asked to give consent to contact their partners by signing or thumb-printing a ‘consent to contact partner’ form. Their male partners were then contacted on phone by the research team through the mediation of the health staff. Due to the sensitive nature of the study, mediated access^2^ was arranged by identifying a staff working in the HIV clinics who were provided information about the participant to assist with the location. Forty women were screened for abuse using the abuse assessment screening (AAS) tool. This was a validated tool adapted and modified from McFarlane et al. [33] that assesses physical, sexual and psychological violence among others. Some of the questions on the tool include;

1. Have you ever been emotionally, physically, financially, sexually or otherwise abused by your partner or someone important to you? YES NO
2. Within the last year, have you ever been hit, slapped, kicked, or otherwise physically hurt by your partner? YES NO

If YES, who? (Circle all that apply)

Husband, Ex-Husband, Boyfriend

Of these women, 20 agreed for their partners to be contacted and out of these 11 male partners who took part in the study, the other 9 declined participation with the reasons of fear of police arrest, stigmatisation, and no time to spend for the interviews. Where it was identified that women were abused and did not want to contact their partners themselves even though they agreed to the team contacting their male partners, health staff helped in contacting them. The safeguards to prevent escalation of violence were that the Ghana police were informed, the social welfare office was also involved. Those participants who agreed to participate contacted the research team willingly in the period of one week after the screening was done. The researcher met and explained to the potential participants, the nature of the study and what was required of them. Those potential participants that decided to be part of the study, gave written informed consent and were recruited as participants. Eleven (11) were enlisted through this process.

The two settings in Ghana include both men and women and so men who came to the facility with their female partners or as patients were directly screened for inclusion. The study settings were two HIV clinics in two Hospitals situated in two districts in the Ashanti region of Ghana. Participants were enlisted as part of the study if they satisfied the inclusion criteria and agreed to participate in the study. They were recruited by explaining the nature of the study and the requirements as well as obtaining written informed consent. Fifteen (15) men were screened and out of these, 7 agreed and were enlisted for the study making a total of 18 participants. Throughout this study, pseudonyms were used for participants.

### Data collection procedures

Data were collected between March and June 2019. Participants were allowed to choose the date, time and venue suitable to them for conducting the interviews. Individual interviews were conducted by a male interviewer (first author) in English or Twi because these are the languages spoken by the people in the locality. A structured interview guide was used and probing to stimulate free individual expressions of men on their perspectives of perpetrating IPVfollowing disclosure of being HIV seropositive was done. The interviews were audio-recorded with a digital voice recorder and each interview lasted for about 60** **min. The interviews were transcribed verbatim by a bilingual expert in both English and Twi immediately after each interview. All interviews were transcribed into English for analysis purposes and the researcher then checked all interviews to ensure that the meaning was not lost in the translation. Field notes were written on participants’ non-verbal behaviours that were observed during the interview.

### Data management and analysis

The data analysis was managed with NVivo software version 12. Transcripts were read several times to fully understand men’s perspectives with regards to perpetration of violence on their HIV-positive female intimate partners following disclosure of HIV seropositive status. The transcripts were coded and similar codes were grouped. The first and second authors coded the data independently and differences were discussed for a consensus on the most appropriate code for a piece of data reached. Subsequently, themes and sub-themes generated were discussed among the three research team members and any discrepancies were resolved by going back to the raw data to ensure that the themes and sub-themes accurately represented the world of the men who participated in the study.

### Rigour

We made sure that trustworthiness was kept during the whole study collection period [34]. To ensure this, only one member collected all the data making sure that the same pattern of the questioning technique was maintained. Data collection and analysis were done concurrently to ensure both data saturation and the discovery of developing themes. Inter-coder agreements were also done with two independent researchers. Member checking [35,36] was done by returning verbatim transcriptions to participants to evaluate and ensure themes and sub-themes produced during the concurrent analysis were the reflections of the participants’ views of their experiences of perpetrating IPV following their female partners’ disclosure of being HIV seropositive. The participants reviewed the transcripts as a true picture of their views on the second interview after transcription was done. Data analysis was also supervised by experienced qualitative researchers. An audit trail was also kept in detail and verbatim quotes, as well as peer debriefing, gave an adequate context that can enhance the application of findings in similar settings.

### Ethical clearance

Ethical clearance was received from all authorities including the Kwame University of Science and Technology’s ethics research committee (CHRPE/AP/572/18) and the South African national research committee (BREC REF: BF629/18). Signed informed consent forms were obtained from the participants and they voluntarily participated.

## Results

The ages of the participants were between 31 and 55** **years old. The majority of the participants 10(56%) had education up to the level of Junior High School (JHS)/Middle School, 4(22%) had basic education at the primary school level while secondary and tertiary education had 2(11%) and 2(11%) respectively. This is representative of people who utilise public HIV clinics in Ghana. Most of the Participants 14(78%) were Christians while 3(17%) were Muslims and 1(6%) was traditional believer. Traditional believers are those individuals who believe in the African traditional religion. These are the three major religious groups in the country. Most participants 7(39%) were still married to their HIV seropositive partners, 6(33%) had recently divorced their HIV seropositive partners following disclosure of seropositive HIV status, while 2(11%) participants were not divorced but were separated or estranged from their partners at the time of data collection as a result of the seropositive HIV status. One participant had two wives and another participant had divorced the wife who disclosed to him that she was HIV positive and married another one. About, 12 (56%) participants were traders or businessmen while 3(17%) were farmers, and 2(11%) were pub operators. Of the 18 participants, 13 (72%) were HIV positive while 5 (28%) were tested HIV negative. The details of the background information are as seen in Table 1 below.

**Table 1.** Background information.

		Frequency	Percentage (%)
Age	Less than 40 years	13	72
	41 − 50 years	3	17
	51 years and above	2	11
Age of spouse	Less than 40 years	16	89
	41 − 50 years	1	6
	51 years and above	1	6
Educational level	Primary	4	22
	Junior High School (JHS)/middle	10	56
	Senior High School (SHS)	2	11
	Tertiary	2	11
Religion	Christian	15	83
	Muslim	3	17
	African Traditional believer	1	6
Occupation	Trader/business	12	67
	Farmer	3	17
	Beer bar operator	2	11
	unemployed	1	6
Marital status	Married	7	39
	Divorced	8	44
	Separated	2	11
	Widow	1	6
Length of marriage	Less than 1 year	5	28
	1–2 years	5	28
	3–4 years	6	33
	5 years and above	2	11
HIV status	Negative	5	28
	positive	13	72

Five main themes were actively developed from the analysed data as the findings of the study. These themes were perspectives on perpetration of emotional, psychological and verbal abuse; perspectives on no engagement in sexual activities; perspectives on perpetration of social isolation; perspectives on perpetration of financial abuse and perspectives on escalated perpetration of physical abuse. Some of these themes had sub-themes. See Table 2 for more information.

**Table 2.** Main themes and sub-themes.

Number	Theme	Sub-theme
Theme 1	Perspectives on emotional, psychological, verbal and spiritual abuse	Belittling/Mocking/Ignoring Instilling Fear in partner Discriminating/Stigmatising Against partner Verbal abuse/Insulting partner Spiritual deprivation
Theme 2	Perspectives on no engagement in sexual activity	Sexual deprivation due to loss of sexual attraction Sexual deprivation as a form of punishment Sexual deprivation out of fear of HIV infection
Theme 3	Perspectives on social isolation	Accusing partner of bringing bad disease Distancing himself from partner/stopped doing things together Divorcing/separating from partner
Theme 4	Perspectives on financial abuse	
Theme 5	Perspectives on escalated physical abuse	

### Theme 1: perspectives on emotional, psychological, verbal and spiritual abuse

This theme came from data related to how the participants reacted to the disclosure by perpetrating intimate partner violence which affected their partners emotionally, psychologically and spiritually. Five sub-themes that were associated with the perpetration of the emotional, psychological, verbal and spiritual abuse included; Belittling/Mocking/Ignoring partner, Instilling fear in partner/discriminating against partner, verbally and spiritually abusing partner.

#### Belittling/mocking/ignoring partner

Some of the participants stated that they intentionally did hurtful things to their partners to make them feel inferior just because their female partners were HIV positive. These behaviours included belittling their partners, telling them they have small brains and could not take decisions on their own and needed to be directed or followed round to help them out as demonstrated in the following extract below.

I asked her to accompany me for the tenants to also see that she was having an extramarital relationship. I told her that her mind is small. She can be deceived by other men and she will forget that she has this condition (Kojo, separated).

Some of the participants also handled their female partners as if they were children because they were HIV positive. Participants did this because they felt it is only children who must learn good and moral behaviour from adults and if their female partners had gotten infected from HIV, then they behaved as immature people who should not be treated as adults but as children. This ideation formed the basis for the violence perpetration on their female partners. In Ghana, gender norms have it that women should obey their male partners without questions [17,18]. Also, it is considered that when a woman behaves irrationally she is considered not mature enough as an adult and so such women are like a child who has not yet grown and matured to reason out things for himself or herself. As a result of this notion, wives are beaten when they go wrong or considered to offend the husband or intimate partner [37–40]. This is an extract from a participant:

I used to handle her like a child by directing her as to what to do with her daily activities such as what to cook, where to go and how to bath in order not to smell. She should have known that such behaviours are for children (Naba, separated).

As a result of their female partner’s HIV status, participants narrated how they forced them to do things against their will. They treated them as though they were no more people who could reason and do things for themselves. This type of action in the views of some participants might have made their female partners feel useless and belittled. However, some of the participants regretted their actions and showed remorse with sad facial expressions during the interviews. Some of them had to plead with the researchers to help them locate their divorced partners for them. They did this with sad and tearful facial expressions. This is what another participant said:

I usually made her feel small and useless by forcing her to do what I want her to do for me and not what she thinks is good (Ojo, divorced).

Again, participants described how they mocked and ignored their female partners. They made fun of them and did not give them attention as they used to do when their female partners were not diagnosed as being HIV positive. One of the participants said:

I started to ignore her when she spoke to me… I wouldn’t pay attention to her if she was talking to me….I mocked at her by making fun of her (Baba, divorced).

Some of the participants intentionally ignored and mocked at their female partners when they informed them of their HIV-positive status. One participant narrated:

I started to ignore her…… I didn’t see her as worthy of my attention… I started making fun of her (Dwomo, married).

#### Instilling fear in partner

Some participants were of the view that women needed to fear them as men and as such had to instil fear into them for the women to obey and fear the men. In line with this, participants used some verbal or non-verbal means of communicating this to the participants by either not communicating with them or looking at them scornfully and even ignoring their treatment which is a way of telling them that they have no time for them. This was always done with hatred. This was what one participant said:

I have to let her fear me because I am a man. I looked at her so scornfully and felt I should kill her because she needs to know what I want her to do for me and that is to respect and obey me (Kojo, separated).

Some participants shouted at female partners to instil fear in them. The hostile behaviour by participants when their female partners disclosed their HIV status to them was so serious that it made the victims of this violence which was just a reaction to the disclosure and failure to contain the effect of the disclosure fear them. It made victims depressed, withdrawn and they distanced themselves from the perpetrators. This is how a participant puts it.

I shouted at her any time she asked me for something. This made her fear me a lot. Any time I came home she was highly uncomfortable and she would find a place to sit far away from me (Dwomo, married).

Some of the participants were already abusive to their partners even before the disclosure of seropositive HIV status but the abuse was intentionally intensified following the disclosure as indicated below:

It was worse when she told me that she was infected with HIV … I shouted at her and intimidated her all the time. I think she only felt happy at times when I was not at home. She only felt free at times when I travelled. (Salifu, divorced).

#### Discriminating/stigmatizing against partner

As a result of the disclosure, participants narrated how they discriminated and stigmatised against their female partners. They did this because they fear their female victims would infect them. They would even refuse to touch anything of them. This is how one participant stated his:

I stopped using the sponge that we were using together because I think I will get infected when I use it… I don’t even want to touch her items because I am afraid she will infect me (Ojo, divorced).

Other participants described how they used to stigmatise their partners when they were informed of the HIV-positive status of their female partners. They did this by refusing to share things they otherwise were sharing for fear of being infected by the female partners. One of the participants said this:

We were using a reusable shaving stick (the one that you can change the blade) together but I stopped using it after she informed me that she was HIV positive… I stopped sharing things with her because I think I will get infected too… I was not willing to even touch her (Kojo, Separated).

Some of the participants also narrated how they stigmatised against their female partners. They would not bathe with them as they used to do nor eat with them again. This behaviour made the female partner emotionally hurt and traumatised. The irony of it is that some of these participants were also HIV positive when they went to do the test but they insisted it was the female partners who got infected first and then infected them. One participant said:

When she informed me of her HIV status, I didn’t want her to touch my things, bathe with me or even prepare food for me because of the condition (Yakubu, married, HIV positive).

One participant also said:

I didn’t bathe nor eat with her after she disclosed to me that she was HIV positive (Adama, widowed).

#### Verbal abuse/insulting partner

Most of the participants showed their displeasure to their partners after disclosure to them that they were HIV positive by perpetrating verbal abuse in the form of insults. Some of the participants reacted to the disclosure by shouting at them and even sacking them from their homes for acquiring the infection. The majority of the participants insulted their partners when they informed them that they were HIV positive. This trend was very pervasive among participants and those who perpetrated these verbal attacks also perpetrated other forms of abuse. One of the participants insulted his partner this way.

When she informed me of her HIV status, I insulted her… She began to be afraid to talk to me because I would respond by shouting and insulting her (Adama, widowed).

Some participants felt that they were condemned to death when they got to know that their female partners were HIV positive and described them as being possessed by evil spirits. One of the participants saw his partner as being possessed by evil spirits and even added that she was a murderer by merely being HIV positive and as a result insulted her this way:

When I was told that she was HIV positive, I started insulting her…She was a devil to me and I felt she was a murderer (Kojo, separated).

In a related style of insults, some of the participants felt that if one gets infected with HIV, it was a disgrace and shame, not only to the couple but also to the family and community. This participant rained insults on his partner this way:

I started insulting her when she informed me that she was HIV positive…So I used to insult her for bringing family embarrassment and shame …I insulted her a lot (Yakubu, married).

#### Spiritual abuse

Most of the participants reacted to the information about HIV + infection status by refusing to pray with their partners because they felt there was no more hope and that it was not necessary to pray with somebody who had brought condemnation to the family. One of the participants said:

After I was told that she was HIV positive, we stopped praying together (Adama, widowed).

Again, most of the participants stopped praying with their female partners because they felt they too were going to die with their female partners and so there was no time to pray with them. This participant said:

For praying, I stopped praying with her after the disclosure. We used to pray together but when I got to know that she was HIV positive, there was no time to pray with her (Papa, married).

Other participants also made statements in similar directions which meant that the spiritual life of these participants, especially concerning their partners, was no more an issue to them. They did not care about their female partners’ spiritual wellbeing. These two participants said:

After her HIV status disclosure, I don’t pray with her anymore (Sarpong, divorced).

When she informed me that she was HIV positive, I stopped praying with her….I didn’t pray with her anymore (Yakubu, married).

### Theme 2: perspectives on no engagement of sexual activity

#### Sexual deprivation due to loss of sexual attraction

The participants narrated how they punished their female partners by refusing to engage in physical intimacy with them. For instance, some of them stated that when their female partners were not known to be HIV positive, they, the men, used to force them to sleep with them but after the disclosure, they stopped engaging them in any physical intimacy. The refusal to have any sexual intimacy with them was a result of bad feelings towards them due to the disclosure of the female partner’s seropositive HIV status. They further indicated how they previously had forced sex with their wives but stopped this following disclosure of the HIV-positive status. This they viewed as a loss of sexual attraction due to physical deterioration. One of the participants stated his perspective this way:

At first, I sometimes had forced sex with her before the diagnosis but now I don’t even like having sex with her because of HIV… I drove her out of my house and she went to stay in Obuasi for about 1 year and because of the situation, I didn’t want to date any lady. I didn’t date anyone and so when she came back I forced her to stay with me and we came together again and started having sex… As for sexual affairs, with counseling we now have sex but not as before because I still feel bad towards her and don’t have any sexual attraction towards her anymore (Kojo, separated).

Some of the participants who were also HIV positive also stopped having sexual intimacy with their partners when they were informed of their female partners’ HIV-positive status.

After she got the infection, I don’t have sexual intercourse with her anymore. I have not slept with her… About the sexual relationship with my wife, I didn’t want to have sexual intercourse with her after she was diagnosed with HIV (Kweku, married, HIV positive).

Sex deprivation was perpetrated by most participants in the study. Two of the participants narrated their experience this way:

After she informed me about her HIV status, I stopped having sex with her again (Papa, married).

I refused to have sex with her from the day she told me about her HIV status (Yakubu, married).

One participant put his views this way:

As for sexual affairs, at first, when she informed me about her status, I stopped having any sexual relationship with her but with counseling, we now have sexual relationships but not as before the diagnosis because I still feel bad towards her and don’t have that feeling anymore. The first time we realized it, according to sources from the nurses, they said we should use a condom for the meantime for about 5 months and then stop using the condom after 5 months of taking the medication, we can then have sex but there are changes now. Some of the changes are that I don’t have sexual feelings for her anymore and it looks like I hate her still. I feel she is now dating a different person as well (Kojo, separated).

#### Sexual deprivation as a form of punishment

Participants who associated their sexual deprivation for their partners following HIV-positive status disclosure use this strategy as a form of punishment because they felt their partners had infected them with HIV. One participant stated that from the date she informed him of her status, he refused to sleep with her. This is what he said:

From the day she informed me about her HIV status, I stopped having sexual intimacy with her as a way of also telling her she infected me with the disease (Naba, separated).

Some participants’ sexual desire towards their female partners was reduced as a result of the status disclosure of their partners. One participant claimed that:

We used to have sex weekly but after the diagnosis, it reduced to once a month (Kwame, divorced).

#### Sexual deprivation out of fear of HIV infection

Most participants whether HIV positive or not, refused to have sex with their partners because of being infected by them. One of the participants alluded to this as follows:

After she informed me of her HIV status, things have changed. The things that have changed are that I don’t have affection for her anymore. I don’t even want to touch her and her items because I am afraid she will infect me with AIDS too (Dwomo, married).

### Theme 3: perspectives on social isolation

Participants reacted to the seropositive HIV disclosure by perpetrating violence in the form of social isolation by accusing the women of infecting and bringing bad disease to the family. Three sub-themes that were associated with perpetration of social isolation were actively developed from this theme and included; Accusation of infection/bringing bad disease, Distancing and stigmatising against the woman.

#### Accusing partner of bringing bad disease

Participants accused their partners of infecting them with HIV. This gave way for them to unleash violence on their partners. Accusing a partner wrongly is abuse in itself. This is what one participant said.

After she informed me of her HIV status, I was really sad. I was very angry. I accused her of infecting me with the condition (Adamu, divorced, HIV positive).

There was also the feeling that their female partners had betrayed them because the infection is a problem brought by their female partners and also accused their female partners of infecting them. This is what this participant said:

After she informed me, I accused her of bringing all these problems and also accused her of betraying me by infecting me with this disease (Kofi, divorced, HIV negative).

In a related development, some participants accused their female partners of getting the disease first and infecting them and so one participant described his partner as a disgrace and accused her of killing him. As a result, he divorced his wife. This was what he said.

After she informed me about her HIV status, I accused her of getting the disease and infecting me with it… So I accused her of disgracing me and the family and also accused her of wanting to kill me and indeed has done it (Kojo, separated, HIV positive).

Another participant abused his wife because he felt that his wife had brought shame to the family:

Whenever we quarrelled, I told her she was the one who brought the shame and didn’t deserve to be my wife, she killed me and my generation because how am I going to take care of the child now that I was going to die? (Dwomo, married).

Yakubu also put his this way:

When I was told about the condition, I told her she brought a bad disease to the family (Yakubu, married).

#### Distancing himself from partner/stopped doing things together

Participants reacted to the disclosure of seropositive HIV status by distancing themselves from their partners and some also stopped doing things with them. This is what one participant said:

After the disclosure, I refused to eat her food because I was disgusted by her… I don’t even want to touch her items because I am afraid she will infect me… I told her to stay away from me and I didn’t want to see her as her presence angers me… Sometimes I felt like running away from the house because the woman had become like a monster and so I feared her so much as to be close to her (Dwomo, married).

Some of the participants also ran away from their partners upon hearing from them that they were HIV positive. A participant stopped helping his partner after the disclosure. He said:

After the HIV status disclosure, the household chores were now her duty without my assistance because I felt let down by my wife… I didn’t help her anytime she needed anything (Yakubu, married).

One participant also stopped doing things together with his partner when he was informed that his partner was HIV positive. He said it this way:

Before the status disclosure, we used to do everything together but after the disclosure, it is not like that again, I stopped doing things with her…I won’t even be happy interacting with her again (Sarpong, divorced).

#### Divorcing/separating from partner

Some of the participants stated that they were so sad and angry that they perpetrated violence against their partners by asking them to leave their homes in the form of divorce or separation. One of them did this by separating from his partner but later brought her back into the marriage. This is what he said.

I started shouting and sacking her from the house when she told me of her condition… I asked her to leave my house (Dwomo, married).

A participant divorced his female partner because he was angry and also because he felt his partner had infected him with the disease or because he felt she had done something very bad to him by getting infected with the disease. He said:

I told her that she should leave my house when she told me she had this condition. I was very angry and told her if this is what she has done to me, then she should go her way and let me also go my way (Adamu, divorced).

Another participant expressed his frustration and anger this way:

When I was informed about it, I shouted at her to leave my house…. she should leave…I was very angry and shouted at her that she should go her way and let me also go my way (Yaya, divorced).

### Theme 4: perspectives on financial abuse

Most of the participants reacted to the disclosure of their HIV-positive partners by depriving them of financial assistance. This was in the form of financial deprivation. This was clearly stated when this participant said:

I stopped giving her money after I heard of the disease… I didn't support her financially because she wasn’t worth it anymore. I used to give her housekeeping money before the diagnosis but after the diagnosis, it even pained me to give her money. I stopped giving her money… I didn't give her money because of the disease, I also didn’t give her money to buy food or drink (Kwame, divorced).

Another participant, in trying to prove his masculinity concerning financial commitment, had this to say:

As a man, it is your responsibility to do everything when you are living together with your wife, so I was doing everything for her but after the diagnosis that was when my money stopped going to her side like that (Kojo, separated).

Again, a participant who had divorced the wife because of the diagnosis shared this about finances. He said:

I no longer support her financially because she is not worth it… It even pains me to give her money to buy food. I stopped giving her money at all (Sarpong, divorced).

Participants also had some issues with financial deprivation. They felt that if a partner is not faithful and has infected them, she should not be supported financially. This view was vividly captured when one of them said:

I felt if she could infect me with the virus then she should also be able to get money from elsewhere to take care of herself. As a result, she went away but called me on the phone and asked that I should send her money but I said to her I can’t send her the money because she has infected me with the disease (Kojo, separated).

Some of the participants stated that they refused to give financial assistance to their partners without giving reasons and the reason that can be inferred is fear of the infection. This is what one of the participants said:

After she informed me of her HIV status, I didn’t give her money again (Adama, widowed).

### Theme 5: perspectives on escalated physical abuse

Some of the participants used to beat their partners but the perpetration of physical abuse escalated when the partners disclosed their HIV-positive statuses to them. This is what one participant said:

Previously, I used to fight with her by occasionally slapping her when she did something wrong but this escalated to beatings almost daily when I was told of her condition (Kojo, separated).

From some of the accounts, participants beat their partners after disclosure to the seropositive HIV statuses of their partners. These are some of the physical assaults as captured in this study.

I had beaten her before the diagnosis of the condition…but it has escalated to beating her physically daily, sometimes throwing objects at her since I was told of her condition and whenever I was angry and frustrated (Baba, divorced)

I beat her once when she made me very angry before the diagnosis but this escalated when she told me of her condition (Naba, 36yrs old, separated).

Another participant also reacted to the HIV-positive status disclosure this way.

I have beaten her before but that was a very long time ago but it escalated when she informed me of her HIV status (Salifu, divorced).

## Discussion

The study explored and described men’s perspectives of perpetrating violence following their female intimate relations’ information about the HIV + infection in Ghana. The results revealed that participants reported having perpetrated different forms of IPV on their partners after they had informed them of their HIV + infection status. Interview participants’ perspectives on the perpetration of IPV after the disclosure of the HIV + status of the female partners was the purpose of this study. Participants narrated their experiences after their partners informed them that they were HIV positive. This led to violence perpetrated against the women in the form of emotional/psychological/verbal/spiritual abuses, no engagement in sexual activity, social isolation, financial abuses, and physical abuses. These five themes were actively developed from participants’ narratives.

Belittling, mocking at, and ignoring participants’ female partners after the disclosure of their HIV statuses in the views of the interview participants might have caused emotional and psychological problems to their partners. The findings reveal that some participants narrated of having perpetrated emotional and psychological abuses and socially isolated themselves from their partners when they learned of their HIV-positive status. Verbal abuses and refusing to engage the partners in prayers were some of the emotional and psychological problems as narrated by participants. Similar findings have been reported in other studies where HIV-positive participants reported that they experienced emotional abuse and abandonment following disclosure of HIV-positive status to their partners [1,38,41]. Due to these emotional and psychological issues, interview participants believed that their behaviour made the female partners fear disclosing their statuses to anybody. Fear to disclose one’s HIV status [42] causes emotional and psychological trauma as found in this study. One of the experiences, as narrated by the participants, is coming home late as a way of reacting to the disclosure and eventually leaving the relationship which constituted emotional abuse to their female partners. This led to themselves socially isolated following the disclosure of positive HIV status which according to them had made their partners psychologically traumatising [18,43,44]. IPV is a complex health problem especially when it happens in families in Ghana [42]. The diagnosis of HIV is in itself traumatising. These findings reveal that some participants either separated or divorced their partners when they learned of the partner’s HIV status because of fear of being infected by the virus or because of the stigma associated with being in an intimate relationship with a person living with HIV.

Again, discriminating and stigmatising against women were identified as some of the abuses which were consistent with Angula et al. [45] who found that external stigma was high of persons who are HIV positive and discrimination based on their HIV positive status. Angula et al. [45] in their study found that stigma manifests in different ways and revealed verbal abuse, social isolation, negative self-perception, and household stigma experienced by people living with HIV/AIDS (PLWHA). Furthermore, health care workers reported that stigma was associated with care providers for people living with HIV/AIDS. They found similar results that collaborate with this study that stigma exists in the form of fear of contagion and workplace stigma. Fear, ignorance, and discrimination regarding HIV continue to exact profound human costs including, in the worst forms, abusive treatment, and violence being perpetrated especially by male partners [46].

Verbal abuses such as insulting the victims were revealed by participants in this study which in the views of interview participants, caused emotional and psychological problems to the intimate partners. Previous studies [47] have collaborated on these findings. Some of the participants narrated how they reacted to the disclosure by refusing to pray with the partners as a result of a loss of hope and the feeling that prayer was of no essence since having HIV infection was condemned to death. It was also a form of abomination to the family.

No engagement in sexual activities was identified to be one of the themes that emerged. These, in the views of participants, were refusal to have physical intimate relations, sex deprivation as a result of a loss of sexual attraction and bad feelings. This was done to serve as a form of punishment for bringing the disease to the family and also the fear of contracting the disease. These findings were collaborated by previous studies [39,41]. In contrast, unwanted sexual advances are most often associated with rape and sexual violence [48–50]. However, in this study, the perpetration of sexual violence, including sexual deprivation was reported as a form of punishment when the partner disclosed to her male partner that she was HIV positive. The perpetration of sexual deprivation was also reported to be associated with the fear of being infected as well as a feeling of anger and betrayal. Sex is a physiological need. Some of the participants also felt that their partners were in extramarital relations and as a result, they got the disease. This feeling made participants not engage in sexual intimacy with them because they felt it was through such behaviour that their female partners got infected with the virus.

In the views of some of the participants of this study, they refused to have sex or sleep with their partners because they felt their partners had infected them with HIV. This was a way of showing their male dominance over their female counterparts. They described them in derogatory terms and saw them as being unfaithful because they believed their partners got the infection from prostitution or sex with other men. These findings are in line with the literature [18,43,44,51] which found different forms of sexual offences and abuses such as refusal to have sex after HIV positive status disclosure, refusal to use a condom, forced sex, denial of sex among others.

Money is the main source of livelihood for the family and so when it is reduced, this can become an issue among intimate partners within the household. The woman in the Ghanaian context is the one responsible for managing the finances in the home and making sure there is money for the meals of the family members including the husband or partner. The man is the provider of the family finances. If for one reason or the other, the provider refuses to provide money, then the family finances will be in jeopardy. Most of the participants of this study expressed their experiences of reducing finances provided to their relations after the HIV + status was disclosed. This was reported as a form of punishment because the woman was no longer regarded as worthy enough to be given money or felt that if a partner is not faithful and has infected them, she should not be supported financially. Several studies have identified financial deprivation, reduction or restriction of finances, and the refusal to support their partner financially following disclosure of seropositive HIV status elsewhere [18,51] which supports the study findings.

The findings reveal the perpetration of escalated physical abuse following the partner’s disclosure of seropositive HIV statuses. Similar findings were reported that disclosing the HIV status increased the probability of perpetrating violence against HIV + women by their male partners [21]. Women infected with HIV were significantly more likely to have had a physically violent male partner at some time and to have experienced physical and/or sexual violence with a current partner [16].

Even though some participants were also diagnosed HIV positive, they still accused their female partners of bringing the disease to them and disgracing the families. Some of these participants divorced or separated from them because of the infection. Other participants reported having stopped sharing certain items with their partners because of fear that they also would be infected. We view this pattern of behaviour as intentional and discriminatory against the HIV-positive partner. These findings are similar to Simbayi et al. [52] who found forty percent of HIV/AIDS persons who had experienced discrimination as a result of the infection and 1in 5 people who lost their accommodation and/or job due to the HIV infection.

## Conclusion

The participants were males who perpetrated IPV against their female partners who were HIV positive. The participants’ intimate partners who were the victims were all HIV seropositive. Some of the participants were also HIV positive; so they were either concordant or discordant partners. Participants narrated having visited violence on their female intimate partners. The results revealed that there was violence against female victims after the disclosure of the infection statuses. Further studies should consider both perpetrators and victims as well as that of the female partners who are HIV positive. The researchers recommend screening of all newly diagnosed HIV-positive women for abuse as an additional prevention strategy for IPV associated with disclosure of positive HIV status.

## Limitations and strengths

The limitation of this study was that it was only men who perpetrated IPV against their female HIV-positive partners. The strength of this study was that the study helped men to reflect on what they were doing to their partners and acknowledge that it was IPV. It was recommended that guidelines be developed to detect and help both victims and perpetrators of IPV in the context of HIV couples.

## Policy implications

This study is expected to also inform decision-makers in health about what to do to improve the relationship among HIV-positive women and their partners as well as health professionals. It is also to improve their knowledge of intimate partner violence against HIV-positive women by their male intimate partners. This study will also serve as a guiding framework for better communication in health institutions and also be of benefit to the health field since intimate partner violence especially against HIV infected women by men is paramount to health care. Another contribution may be specific to the Ministry of Health in Ghana since most of such IPV studies are in the Social Sciences, not in health which can be of help in the formulation of policies on IPV.

## Data Availability

The datasets used and analysed during the study are available from the corresponding author on reasonable request.

## Notes

1 Seropositive HIV Status is when an individual is diagnosed of having the HIV infection.

2 This means a staff assisting the researchers to the health facilities or clinics.

## Abbreviations

AAS: Abuse assessment screening
HIV: Human Immunodeficiency Virus
IPV: intimate partner violence
PLWHA: People living with HIV/AIDS
PLWHIV: people living with HIV
SSA: Sub-Saharan Africa
